# Impact of bundle for central line associated bloodstream infections prevention

**DOI:** 10.1186/1753-6561-5-S6-O11

**Published:** 2011-06-29

**Authors:** RE Quirós, L Fabbro, A Novau

**Affiliations:** 1 Prevention and Infection Control Department, Hospital Universitario Austral, Pilar, Argentina

## Introduction / objectives

Bundles have been developed to facilitate the application of infection control guidelines. Because in our institution the rates of central line associated bloodstream infections (CL-BSI) were above the international standards it was decided to implement a specific bundle through a multimodal approach.

The aim of this study was to describe the strategy of bundle implementation for prevention of CL-BSI and to estimate their impact.

## Methods

Since Mar’10 the following measures were implemented at the ICUs to prevent CL-BSI: use central venous catheters only if strictly necessary; avoiding the femoral site if possible; hand hygiene with alcohol-gel before insertion; using full-barrier precautions during the insertion of central venous catheters; cleaning the skin with chlorhexidine (2%) and removing unnecessary catheters. The implementation was carried out through the model of "5Es" (Engage, Education, Execution, Evaluation and Encouragement). The rate of CL-BSI during the intervention period (Mar’10-Feb’11) was compared with the average of the 12 months prior to implementation. All costs are expressed in US dollars. For economic impact analysis an attributable cost of U$S 5,500 was used.

## Results

The incidence rate of CL-BSI at the baseline period was 6.84 events per ‰ device-days in comparison with 2.70 events per ‰ device-days during implementation period (RR 0.40; 95% CI 0.22 to 0.69, p<0,01). There are no changes in the utilization ratio between both periods (0.45 [6429/14222] and 0.44 [7025/16077], respectively). During the implementation period the level of adherence rises to more than 90% in all bundle components. While the annual incremental cost to prevent CL-BSI was U$S 28,300, the overall net savings was U$S 130,500.

## Conclusion

The effective implementation of this bundle in our hospital reduced the CL-BSI with a significant net saving.

## Disclosure of interest

None declared.

